# Palliative Resection of Pancreatic Adenocarcinoma

**DOI:** 10.1155/1995/54241

**Published:** 1995

**Authors:** C. D. Johnson

**Affiliations:** University Surgical Unit F level Centre Block Southampton General Hospital Tremona Road Southampton SO16 6YD USA

## Abstract

A survey was carried out by postal questionnaire of the attitudes of British surgeons to pancreatic resection as palliation for ductal adenocarcinoma of the pancreas. Replies from 24 surgeons related to experience in over 700 resections. The incidence of estimated residual local disease after resection was median 12.5 percent, range 0–35 percent. Half(12) of the surgeons felt that pancreatic resection with residual macroscopic disease was justified. Only 3 (12.5 percent) surgeons accepted that palliative resection in the presence of liver metastases was sometimes justifiable. Further evidence is required of improved quality of life after resection before the majority of surgeons will accept palliative resection in the management of pancreatic ductal adenocarcinoma.


*HPB Surgery*, 1995, Vol. 8, pp. 181–183
Reprints available directly from the publisher
Photocopying permitted by license only

© 1995 Harwood Academic Publishers GmbH
Printed in Malaysia

### Palliative Resection of Pancreatic Adenocarcinoma

### A Survey of British Surgeons

C. D. JOHNSON

University Surgical Unit, F level Centre Block Southampton General Hospital Tremona Road Southampton SO16 6YD

A survey was carried out by postal questionnaire of the attitudes of British surgeons to pancreatic resection as palliation for ductal adenocarcinoma of the pancreas. Replies from 24 surgeons related to experience in over 700 resections. The incidence of estimated residual local disease after resection was median 12.5 percent, range 0–35 percent. Half (12) of the surgeons felt that pancreatic resection with residual macroscopic disease was justified. Only 3 (12.5 percent) surgeons accepted that palliative resection in the presence of liver metastases was sometimes justifiable. Further evidence is required of improved quality of life after resection before the majority of surgeons will accept palliative resection in the management of pancreatic ductal adenocarcinoma.

KEY WORDS: Pancreas adenocarcinoma surgical treatment

Resection of pancreatic cancer, even for attempted cure, is rarely successful. Almost always, the patient dies of recurrent disease, but may well have extended survival as a result of the operation. Historically, the mortality of resection was so high that palliative resection, in the presence of irresectable local invasion or distant metastases was never attempted. However since 1980 it has become clear that pancreatic resection can be performed with mortality well under 10%. Many surgeons can achieve operative mortality less than 5%. In 28 reports, containing over 2,000 patients, the overall operative mortality was 6.9%[1,2]. The median (interquartile range) mortality reported by each author was 7.5 (4.5–12) percent. If, as some surgeons believe, resection offers the best form of palliation[3], then these impressively low mortality figures lead us to consider the possibility of palliative resection even in the presence of irresectable local disease or distant metastases.

This paper reports a study of current attitudes amongst British surgeons to palliative resection for pancreatic carcinoma.

### METHOD

A questionnaire was sent to 30 British surgeons known to the author to have an interest in biliary and pancreatic disease; 24 replies were received. No reminder was sent. The questions are shown in Table 1.

The responses were tabulated using the Statistical Package for the Social Sciences (SPSS/Inc, Chicago Ill. USA) on an Amstrad 1610 personal computer.

### RESULTS

All surgeons had experience of pancreatic resection, but one had a policy never to perform resection for adenocarcinoma of the pancreas. A total of 703 resections for pancreatic carcinoma was reported by this group of 24 surgeons. Median individual experience was 20 resections (range 0–140).

In their estimate of the cases in which they had left residual local disease the surgeons reported a median incidence of residual disease of 12.5%. The range was from 0–35%, and six (25%) surgeons claimed they had never left macroscopic residual local disease during resection for pancreatic adenocarcinoma.

The responses to the question of whether palliative resection is appropriate are shown in Figure 1. The initial response of two thirds of the surgeons was that it is never appropriate to perform palliative resection, but when asked specifically to consider local extension, the surgeons were split evenly in their acceptance of the principle of palliative resection. Only three surgeons felt that it could ever be justified to perform pancreatic resection in the

**Table 1** Questionnaire sent to British surgeons to determine their attitudes to palliative resection of pancreatic adenocarcinoma

| |
|---|
| Do you believe it is ever appropriate to perform a palliative resection? |
| Is it appropriate to perform palliative resection with residual local disease? |
| Is it appropriate to perform palliative resection in the presence of liver metastases? |
| How many resections for pancreatic cancer have you carried out? |
| On how many occasions was there residual local disease (obvious to the surgeon at operation)? |
| How many times have you performed a pancreatic resection in the presence of known liver metastases? |
| How long have such patients survived? |

presence of liver metastases. One of these had performed three or four elective palliative resections with post-operative survival ranging from six to 18 months. One surgeon had performed palliative resection in the presence of liver metastases, in order to control haemorrhage from a tumour bleeding into the duodenum. One who approved of palliative resection in some circumstances had never seen a suitable case.

### DISCUSSION

Opinion amongst British surgeons regarding the merit of palliative resection of pancreatic adenocarcinoma varies widely. Some have very strict criteria for resection, and avoid operation whenever the tumour might have spread outside the pancreas. Half the surgeons in this survey are prepared to accept residual macroscopic local invasion if this is discovered during the course of a resection. Palliative resection in the presence of liver metastases is approved by only a small minority.

Palliative resection can only be acceptable 1) if operative mortality is low, and 2) if resection produces better palliation than other treatments. The first criterion has been achieved by most surgeons with an interest in this condition[1,2]. The question of quality of palliation relies entirely on opinion[3]. There are no satisfactory published assessments of quality of life achieved by resection or surgical and other forms of palliation, but a Norwegian study has recorded objective evidence of better quality of life in patients who had resection compared to surgical bypass (Bakkevold, Personal Communication). There is preliminary evidence of longer survival in patients who undergo resection compared with patients who have

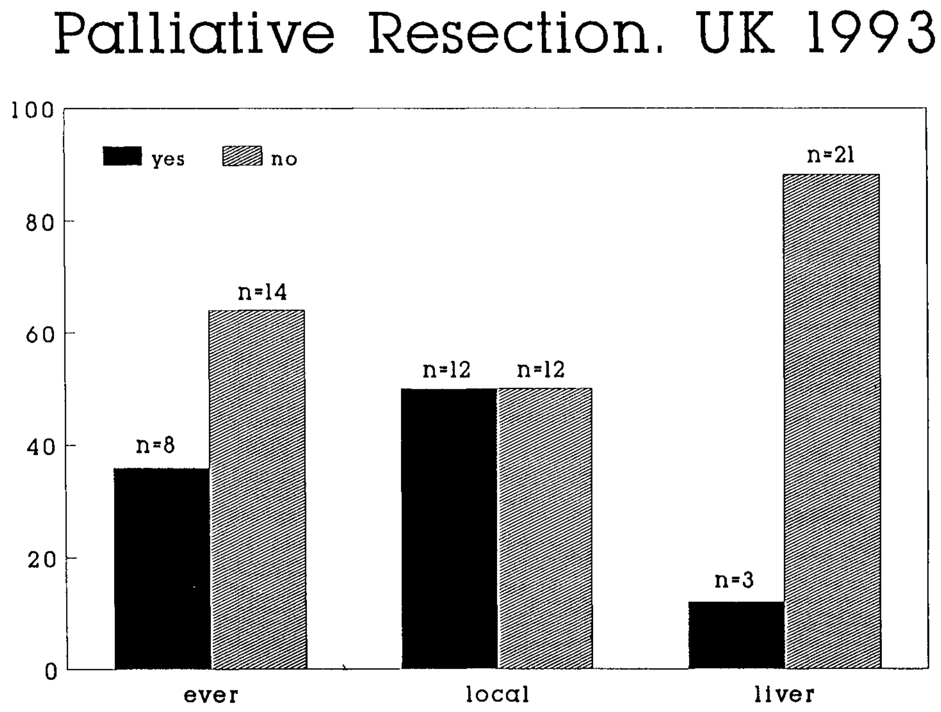

**Figure 1** Response by British surgeons to the question whether palliative resection was ever appropriate, appropriate when there was residual local disease, and appropriate in the presence of liver metastases.

similar stage tumours who did not have a resection[4], and it is reasonable to suppose that tumour debulking might prolong survival.

Palliative resection leaving residual local disease might be considered in conjunction with postoperative adjuvant therapy. Postoperative radiotherapy with 5FU as a radiosensitiser, followed by weekly 5FU, has been shown to increase postoperative survival from 20% to 40% at two years[5]. A similar regimen produced responses in a significant number of patients with irresectable pancreatic cancer[6], so it is reasonable to suppose that palliative resection followed by adjuvant therapy could give effective local control in some patients. However there are no published data to support this supposition.

Pancreatectomy for ductal or adenocarcinoma in the presence of liver metastases is rarely undertaken. In addition to the cases mentioned here, Jeekel (Personal Communication) has performed such a resection in three patients. One patient is still alive more than two years after operation. In Mannheim, a small number of combined pancreatic and liver resections have been performed for this type of case, but the outcome is not clear[7].

In conclusion it now seems that palliative resection is a feasible option in the management of pancreatic cancer. The exact definition of palliative resection is not clear, because even after so called curative resection the vast majority of patients die of tumour recurrence. The majority of British surgeons do not yet accept the concept of palliative resection to improve symptoms. Evidence of improved quality of life is lacking and studies are urgently needed to evaluate this in patients undergoing surgical and other treatments for pancreatic cancer. Resection for pancreatic carcinoma with liver metastases is accepted only by a small number of surgeons and then only in very carefully selected patients with minimal tumour burden. Improvements in adjuvant therapy are required, to match improvements already seen in operative mortality, before palliative resection will be widely accepted.

### ACKNOWLEDGEMENT

I am grateful to my colleagues who returned the questionnaires:

| | |
|---|---|
| Mr D Alderson | Bristol |
| Mr M Cooper | Exeter |
| Mr J Doran | Nottingham |
| Mr D Finnis | Salisbury |
| Mr D Fossard | Leicester |
| Mr R Hall | Derby |
| Mr P Houghton | Torbay |
| Mr JM Kelly | Portsmouth |
| Mr R Kennedy | Yeovil |
| Mr A Kingsnorth | Liverpool |
| Mr McCloy | Manchester |
| Mr J Neoptolemos | Birmingham |
| Mr J Pain | Poole |
| Mr G Poston | Liverpool |
| Mr M Puntis | Cardiff |
| Mr B Rees | Cardiff |
| Mr M Rees | Basingstoke |
| Mr RGC Russell | Middlesex |
| Mr WEG Thomas | Sheffield |
| Mr JN Thompson | London |
| Mr J Trapnell | Bournemouth |
| Prof RCN Williamson | London |

### REFERENCES

1. Johnson, C. D. (1991) Why resect pancreatic cancer? In Pancreatic Disease: *Progress and Prospects*. Eds Johnson, CD. Imrie, C. W. London. Springer Verlag. pp. 97–102.
2. Johnson, C. D. (1993) Pancreaticogastrostomy after resection of the pancreatic head. In: *Standards in pancreatic surgery*. (Eds) Beger H. G., Buchler, M., Malfertheiner, P., Berlin, Springer Verlag, pp. 663–75.
3. Trede, M. (1987) Treatment of pancreatic carcinoma: the surgeon's dilemma. *Br. J. Surg.*, **74**, 79–80.
4. Chandiramini, V. A., Theis, B. A., Russell, R. G. C. (1990) Role of resection in the management of pancreatic cancers. *Gut.*, **31**, A488.
5. Gastrointestinal Tumour Study Group (1987) Further evidence of effective adjuvant combined radiation and chemotherapy following curative resection of pancreatic cancer. *Cancer.*, **59**, 2006–2010.
6. Jeekel, J., Trieurniet-Donker. (1991) Treatment perspectives in locally advanced unresectable pancreatic cancer. *Br. J. Surg.*, **78**, 1332–1334.
7. Trede, M., Schwall, G., Saeger, H. D. (1990) Survival after pancreatoduodenectomy. *Ann. Surg.*, **211**, 447–458.

